# Supplementary material for: Hybrid Models and Biological Model Reduction with PyDSTool
Source: PLoS Comput Biol. 2012 Aug 9;8(8):e1002628. doi: 10.1371/journal.pcbi.1002628 (PMC3415397; doi:10.1371/journal.pcbi.1002628)
Supplement: Text S4 — Complete source code for the PyDSTool package (version 0.88.120504). Includes API documentation and help files linking to web pages. This file is identical to the current public release on Sourceforge.net. (ZIP) [file pcbi.1002628.s004.zip › PyDSTool/html/PyDSTool.PyCont.Continuation.FoldCurve-class.html]

xml version="1.0" encoding="ascii"?


PyDSTool.PyCont.Continuation.FoldCurve


| Home | Trees | Indices | Help | PyDSTool |
| --- | --- | --- | --- | --- |

Package PyDSTool ::
Package PyCont ::
Module Continuation ::
Class FoldCurve

[hide private]

[frames] | no frames]

# Class FoldCurve

source code

```
  object --+    
           |    
Continuation --+
               |
              FoldCurve
```

---

```
Child of Continuation class that represents curves of limit
points.

Augmented system h(x,a): Uses single bordering on the matrix A
given by

        A = f_{x}(x,a)

    (see class PyCont.TestFunc.BorderMethod)
    (see class PyCont.TestFunc.Fold_Bor)

    The continuation variables are (x,a) with

        x = State variables
        a = Free parameters

    and the continuation curve is defined by

        h(x,a) = { f(x,a) = 0
                 { G(x,a) = 0

Detection of points: BT, ZH, CP (BP points not currently
implemented).
```


|  |  |
| --- | --- |
| Instance Methods | [hide private] |

|  |  |
| --- | --- |
| \_\_init\_\_(self, model, gen, automod, plot, args=None)  x.\_\_init\_\_(...) initializes x; see x.\_\_class\_\_.\_\_doc\_\_ for signature | source code |

|  |  |
| --- | --- |
| update(self, args)  Update parameters for FoldCurve. | source code |

|  |  |
| --- | --- |
| \_createTestFuncs(self)  Creates processors and test functions for FoldCurve class. | source code |

**Inherited from `Continuation`**:
`__copy__`,
`__deepcopy__`,
`__repr__`,
`__str__`,
`backward`,
`cleanLabels`,
`computeEigen`,
`display`,
`exportGeomview`,
`forward`,
`getSpecialPoint`,
`info`,
`reset`,
`testdomain`,
`testdomaingrid`,
`testdomaintangrid`

**Inherited from `Continuation`** (private):
`_Keller`,
`_MoorePenrose`,
`_Natural`,
`_checkForBifPoints`,
`_compute`,
`_curveToPointset`,
`_preTestFunc`,
`_savePointInfo`,
`_system`,
`_systemjac`,
`_systemjac_withpars`,
`_systemjacuser`,
`_systemuser`

**Inherited from `object`**:
`__delattr__`,
`__getattribute__`,
`__hash__`,
`__new__`,
`__reduce__`,
`__reduce_ex__`,
`__setattr__`


|  |  |
| --- | --- |
| Properties | [hide private] |

**Inherited from `object`**:
`__class__`


|  |  |
| --- | --- |
| Method Details | [hide private] |

|  |  |
| --- | --- |
| \_\_init\_\_(self, model, gen, automod, plot, args=None)  *(Constructor)* | source code |

x.\_\_init\_\_(...) initializes x; see x.\_\_class\_\_.\_\_doc\_\_ for
signature

Overrides: object.\_\_init\_\_: *(inherited documentation)*

|  |  |
| --- | --- |
| update(self, args) | source code |

Update parameters for FoldCurve.

Overrides: Continuation.update

|  |  |
| --- | --- |
| \_createTestFuncs(self) | source code |

```
Creates processors and test functions for FoldCurve class.

Note:  In the following list, processors are in
PyCont.Bifpoint and test functions are in PyCont.TestFunc.

Point type (Processor): Test function(s)
----------------------------------------

BT (BTPoint): BT_Fold
ZH (ZHPoint): Hopf_Det (or Hopf_Bor), BT_Fold
CP (CPPoint): CP_Fold
```

Overrides: Continuation.\_createTestFuncs

  


| Home | Trees | Indices | Help | PyDSTool |
| --- | --- | --- | --- | --- |

|  |  |
| --- | --- |
| Generated by Epydoc 3.0.1 on Fri May 4 15:24:07 2012 | http://epydoc.sourceforge.net |
